# Supplementary material for: Content-rich biological network constructed by mining PubMed abstracts
Source: BMC Bioinformatics. 2004 Oct 8;5:147. doi: 10.1186/1471-2105-5-147 (PMC528731; doi:10.1186/1471-2105-5-147)
Supplement: Additional File 2 — The original results of the above study (non-essential files are deleted to keep the file size under the limit set by BMC bioinformatics). [file 1471-2105-5-147-S2.bz2 › chilibotAdditionalFile2/dip20/732ID11983079E18090/html/HSCA_HSCB.html]

 


 **HSCA** and **HSCB** 
  
Found 16 abstracts in PubMed, retrieved 16.  
 

 What does Google say? 
 PDF only 
| .edu only 

---

**Interactive relationship** (e.g. stimulation, inhibition, etc)

**Neutral relationship**- Contributions of the LPPVK motif of the iron sulfur template protein IscU to interactions with the Hsc66  [ **HSCA** ]  Hsc20  [ **HSCB** ]  chaperone system.  Ref: 12871959 J Biol ChemJ Biol Chem,
- Hsc66  [ **HSCA** ]   **HscA**  and Hsc20  [ **HSCB** ]   **HscB**  from Escherchia coli comprise a specialized chaperone system that selectively binds the iron sulfur cluster template protein IscU.  Ref: 12871959 J Biol ChemJ Biol Chem,
- These results indicate that residues in the LPPVK motif are important for IscU interactions with Hsc66  [ **HSCA** ]  but not for the ability of Hsc20  [ **HSCB** ]  to target IscU to Hsc66  [ **HSCA** ] .  Ref: 12871959 J Biol ChemJ Biol Chem,
- The Hsc66  [ **HSCA** ]  Hsc20  [ **HSCB** ]  chaperone system in Escherichia coli chaperone activity and interactions with the DnaK DnaJ grpE system.  Ref: 9852006 J Bacteriol, 1998
- Mapping of the transcription initiation site showed that  **hscA**  was cotranscribed with an upstream dnaJ like gene,  **hscB** .  Ref: 7665466 J Bacteriol, 1995
- Hsc20  [ **HSCB** ]  is a 20 kDa auxiliary protein that functions with the molecular chaperone Hsc66  [ **HSCA** ]  in Escherichia coli.  Ref: 9300502 Protein Sci, 1997
- Interaction of the iron sulfur cluster assembly protein IscU with the Hsc66  [ **HSCA** ]  Hsc20  [ **HSCB** ]  molecular chaperone system of Escherichia coli.  Ref: 10869428 Proc Natl Acad Sci U S A, 2000
- Surface plasmon resonance and isothermal titration calorimetry further showed that IscU and Hsc20  [ **HSCB** ]  form a complex, and Hsc20  [ **HSCB** ]  may thereby aid in the targeting of IscU to Hsc66  [ **HSCA** ] .  Ref: 10869428 Proc Natl Acad Sci U S A, 2000
- The  **hscA**  and  **hscB**  genes of Escherichia coli encode novel chaperone and co chaperone proteins, designated Hsc66  [ **HSCA** ]  and Hsc20  [ **HSCB** ] , respectively.  Ref: 9144776 Protein Sci, 1997
- The results presented here indicate crucial roles for IscU,  **HscB** ,  **HscA** , and Fdx as central components of the assembler machinery and also provide evidence for interactions among them.  Ref: 11432781 J Biochem Tokyo , 2001
- In accord with the previous in vitro binding and kinetic experiments, interactions were observed for the combinations of IscS and IscU, IscU and  **HscB** , IscU and  **HscA** , and  **HscB**  and  **HscA** .  Ref: 11983079 J Biochem Tokyo , 2002
- Hsc20  [ **HSCB** ]  is a 20 kDa J protein that regulates the ATPase activity and peptide binding specificity of Hsc66  [ **HSCA** ] , an hsp70 class molecular chaperone.  Ref: 11124030 J Mol Biol, 2000
- A charge mutant containing the motif sequence of NifU, IscU V102E, did not bind the ATP or ADP states of Hsc66  [ **HSCA** ]  but did bind Hsc20  [ **HSCB** ]  and weakly stimulated Hsc66  [ **HSCA** ]  ATPase in the presence of the co chaperone.  Ref: 12871959 J Biol ChemJ Biol Chem, 2000
- Hsc20  [ **HSCB** ]  also decreased the apparent K m for IscU stimulation of Hsc66  [ **HSCA** ]  ATPase activity, and surface plasmon resonance studies revealed that Hsc20  [ **HSCB** ]  enhances binding of IscU to Hsc66  [ **HSCA** ] .  Ref: 10869428 Proc Natl Acad Sci U S A, 2000
- Thus, Hsc20  [ **HSCB** ] , in addition to its role in the regulation of the ATPase activity of Hsc66  [ **HSCA** ] , may also function as a rigid scaffold to facilitate positioning of the protein substrates targeted to Hsc66  [ **HSCA** ] .  Ref: 11124030 J Mol Biol, 2000

**Non-interactive relationship** (e.g. studied together, co-existance, homology, etc.)

- The high level of constitutive expression and the lack of a major response to temperature changes suggest that Hsc66  [ **HSCA** ]  and Hsc20  [ **HSCB** ]  play an important cellular role s under non stress conditions.  Ref: 9144776 Protein Sci, 1997
- Possible interactions between the Hsc66  [ **HSCA** ]  Hsc20  [ **HSCB** ]  and DnaK DnaJ GrpE chaperone systems were also investigated by measuring the effects of cochaperone proteins on Hsp70 ATPase activities.  Ref: 9852006 J Bacteriol, 1998
- Immunoblot analyses of E. coli cultures using antisera to Hsc66  [ **HSCA** ]  and Hsc20  [ **HSCB** ]  raised in rabbits establish that Hsc66  [ **HSCA** ]  and Hsc20  [ **HSCB** ]  are constitutively expressed at levels corresponding to cell concentration approximately 20 microM and approximately 10 microM, respectively.  Ref: 9144776 Protein Sci, 1997
- Strains with mutations in the iscU,  **hscB** ,  **hscA** , and fdx genes also exhibited conspicuous phenotypical consequences almost identical to one another.  Ref: 11432781 J Biochem Tokyo , 2001
- Thus, the sequential association and dissociation among the IscS, IscU, IscA,  **HscB** ,  **HscA** , Fdx, and ORF3 proteins may be a critical process in the assembly of Fe S clusters.  Ref: 11983079 J Biochem Tokyo , 2002
- Only a single substrate, the Fe S template protein IscU, has been identified for the Hsc66  [ **HSCA** ]  Hsc20  [ **HSCB** ]  system, but the mechanism by which Hsc66  [ **HSCA** ]  selectively binds IscU is unknown.  Ref: 11994302 J Biol Chem, 2002
- These findings suggest that Hsc66  [ **HSCA** ]  and Hsc20  [ **HSCB** ]  comprise a molecular chaperone system similar to the prokaryotic DnaK DnaJ and eukaryotic hsp70 hsp40 systems.  Ref: 9144776 Protein Sci, 1997
- We have overproduced and purified Hsc66  [ **HSCA** ]  and Hsc20  [ **HSCB** ]  in high yield in E. coli and describe their initial characterization including absorbance, fluorescence, and circular dichroism spectra.  Ref: 9144776 Protein Sci, 1997
- Hsc66  [ **HSCA** ]  and Hsc20  [ **HSCB** ]  comprise a specialized chaperone system important for the assembly of iron sulfur clusters in Escherchia coli.  Ref: 11994302 J Biol Chem, 2002
- The proteins encoded by  **hscA**  and  **hscB** , the chaperone HSP66 and the co chaperone HSP20, have been shown to be required for the synthesis of 2Fe 2S clusters on ferredoxin in proteobacteria.  Ref: 11689493 Hum Mol Genet, 2001
- The  **hscB** ,  **hscA** , and fdx genes are also located adjacent to the iscSUA genes in both E. coli and H. influenzae.  Ref: 9582371 J Biol Chem, 1998
- Hsc20  [ **HSCB** ] , unlike DnaJ, did not exhibit intrinsic chaperone activity and appears to function solely as a regulatory cochaperone protein for Hsc66  [ **HSCA** ] .  Ref: 9852006 J Bacteriol, 1998
- Hsc66  [ **HSCA** ] , a stress 70 protein, and Hsc20  [ **HSCB** ] , a J type accessory protein, comprise a newly described Hsp70 type chaperone system in addition to DnaK DnaJ GrpE in Escherichia coli.  Ref: 9852006 J Bacteriol, 1998
- To investigate possible interactions between these systems, we have overproduced and purified the IscU protein from Escherichia coli and have studied its interactions with the  **hscA**  and  **hscB**  gene products Hsc66  [ **HSCA** ]  and Hsc20  [ **HSCB** ] .  Ref: 10869428 Proc Natl Acad Sci U S A, 2000
- These findings are consistent with the role in Fe S biogenesis previously proposed for the bacterial Hsp70 Hsc66  [ **HSCA** ]  and J protein Hsc20  [ **HSCB** ]  that interact with the bacterial Isu homologue IscU.  Ref: 12756240 J Biol Chem< MedlineTA>J Biol Chem, 2000
- Sequence differences between Hsc66  [ **HSCA** ]  and Hsc20  [ **HSCB** ]  compared to other members of this chaperone family suggest that the Hsc66  [ **HSCA** ]  Hsc20  [ **HSCB** ]  system will display different peptide binding specificity.  Ref: 9144776 Protein Sci, 1997
- The order of the genes with established functions was groELS trmE rnpA rpmH dnaA dnaN gyrB atpCDGAH FEB gidA fdx  **hscA**   **hscB**  nifS ilvDC rep trxA rho.  Ref: 9516544 Curr Microbiol, 1998
- Together these results suggest that Hsc66  [ **HSCA** ]  Hsc20  [ **HSCB** ]  and DnaK DnaJ GrpE comprise separate molecular chaperone systems with distinct, nonoverlapping cellular functions.  Ref: 9852006 J Bacteriol, 1998
- Unlike native IscU the synthetic peptide is not bound by Hsc20  [ **HSCB** ]  and does not synergistically stimulate Hsc66  [ **HSCA** ]  ATPase activity with Hsc20  [ **HSCB** ] .  Ref: 11994302 J Biol Chem, 2002
- When ATPase stimulatory activity was assayed in the presence of the co chaperone Hsc20  [ **HSCB** ] , each of the mutants displayed enhanced binding affinity, but the P101A and V102A mutants exhibited decreased ability to maximally simulate Hsc66  [ **HSCA** ]  ATPase.  Ref: 12871959 J Biol ChemJ Biol Chem, 2002
- In 56 available genomes we identified two genes with identical phylogenetic distributions to the frataxin cyaY gene  **hscA**  and  **hscB**  JAC1.  Ref: 11689493 Hum Mol Genet, 2001
- Our results indicate that Hsc66  [ **HSCA** ]  and Hsc20  [ **HSCB** ]  recognize distinct regions of IscU and further suggest that Hsc66  [ **HSCA** ]  will not bind LPPVK motifs with high affinity in vivo unless they are in the context of native IscU and can be directed to Hsc66  [ **HSCA** ]  by Hsc20  [ **HSCB** ] .  Ref: 11994302 J Biol Chem, 2002
- Functional assignment of the ORF2 iscS iscU iscA  **hscB**   **hscA**  fdx ORF3 gene cluster involved in the assembly of Fe S clusters in Escherichia coli.  Ref: 10544286 J Biochem Tokyo , 1999
- JAC1, an ortholog of  **hscB** , and SSQ1, a paralog of  **hscA** , have been shown to be required for iron sulfur cluster assembly in mitochondria of Saccharomyces cerevisiae.  Ref: 11689493 Hum Mol Genet, 2001
- Purified Hsc66  [ **HSCA** ]  exhibits a low intrinsic ATPase activity approximately 0.6 min 1 at 37 degrees C, and Hsc20  [ **HSCB** ]  was found to stimulate this activity up to 3.8 fold with half maximal stimulation at a concentration approximately 5 microM.  Ref: 9144776 Protein Sci, 1997
- Hsc66  [ **HSCA** ]  and Hsc20  [ **HSCB** ] , a new heat shock cognate molecular chaperone system from Escherichia coli.  Ref: 9144776 Protein Sci, 1997
- The E. coli  **hscA**  and  **hscB**  gene products have previously been shown to bear primary sequence identity when respectively compared with the dnaK and dnaJ gene products and have been proposed to be members of a heat shock cognate molecular chaperone system of unknown function.  Ref: 9582371 J Biol Chem, 1998
- Because endogenous substrates for the Hsc66  [ **HSCA** ]  Hsc20  [ **HSCB** ]  system have not yet been identified, we investigated chaperone like activities of Hsc66  [ **HSCA** ]  and Hsc20  [ **HSCB** ]  by their ability to suppress aggregation of denatured model substrate proteins, such as rhodanese, citrate synthase, and luciferase.  Ref: 9852006 J Bacteriol, 1998
- These results establish a direct and specific role for the Hsc66  [ **HSCA** ]  Hsc20  [ **HSCB** ]  chaperone system in functioning with isc gene components for the assembly of iron sulfur cluster proteins.  Ref: 10869428 Proc Natl Acad Sci U S A, 2000
- The authors characterized the transcriptional arrangement of  **hscA** , showing that it forms part of an operon with the upstream  **hscB**  gene,.  Ref: 10846221 Microbiology, 2000
- We also examined coexpression using the fdx gene in the isc gene cluster as a reporter Fd and identified iscS,  **hscB** ,  **hscA** , and ORF3 as being involved in the assembly of the 2Fe 2S cluster in this protein.  Ref: 10544286 J Biochem Tokyo , 1999
- Another feature of the iscSUA gene cluster region from A. vinelandii is that E. coli genes previously designated as  **hscB** ,  **hscA** , and fdx are located immediately downstream from, and are probably co transcribed with, the iscSUA genes.  Ref: 9582371 J Biol Chem, 1998
- IscU and its iron sulfur complex IscU Fe S stimulated the basal steady state ATPase activity of Hsc66  [ **HSCA** ]  weakly in the absence of Hsc20  [ **HSCB** ]  but, in the presence of Hsc20  [ **HSCB** ] , increased the ATPase activity up to 480 fold.  Ref: 10869428 Proc Natl Acad Sci U S A, 2000
